# Editorial: Cancer cell metabolism and tumor microenvironment remodel

**DOI:** 10.3389/fgene.2026.1921001

**Published:** 2026-08-10

**Authors:** Daniela Rodriguez-Perera, Katsuhiro Yoshimura, Johannes Fahrmann, Rongzhang Dou

**Affiliations:** 1 Department of Tumor Pathology, Hamamatsu University School of Medicine, Hamamatsu, Japan; 2 Department of Clinical Cancer Prevention, The University of Texas MD Anderson Cancer Center, Houston, TX, United States

**Keywords:** cancer immune, cell metabolism, microbiome, therapy, tumor micro environment (TME)

## Abstract

**Figure:**
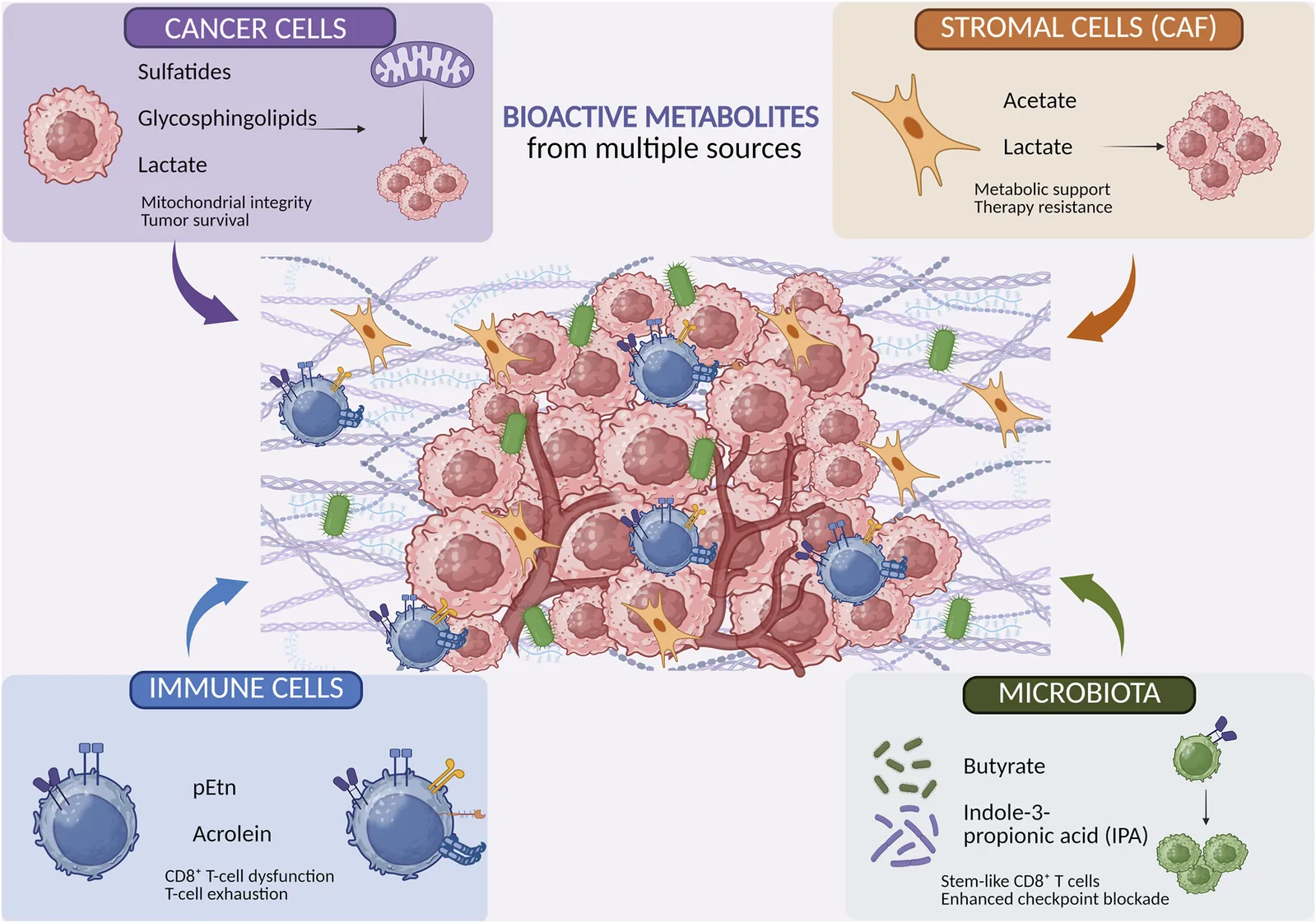

## Introduction

Metabolic reprogramming that supports proliferative energetics is an established hallmark of cancer. Yet, it is becoming evident that metabolic reprogramming extends beyond energetics and that metabolites serve as bioactive molecules that exert biophysical properties to promote cancer cell progression and that aid in shaping the tumor microenvironment (TME). Moreover, these metabolic alterations are not limited to cancer cells, but also reflect the contributions of other cell types within TME, including microbes. In this context, metabolites should be viewed not merely as fuels or metabolic byproducts, but as functional mediators that influence signaling, epigenetic regulation, organelle homeostasis, immune cell fate, and therapeutic response. Here, we shed light on these metabolic adaptations and how this knowledge translates into novel strategies for cancer treatment.

## Emerging metabolic alterations associated with cancer cell survival and proliferation

Metabolic adaptations, primarily characterized in the context of oxidative phosphorylation and aerobic glycolysis, optimize biosynthesis of energy sources and cellular building blocks to support cell proliferation. Paracrine secretion of lactate, certain amino acids, e.g., glutamine, and lipids, e.g., fatty acids, have also been shown to be utilized as alternative sources of ‘fuel’ (Hanahan, 2026).

While less characterized, emerging evidence also implicates biophysical properties of metabolites for maintenance and clearance of intracellular organelles, particularly mitochondria, to support cancer survival and proliferation. To this end, using spatial metabolite imaging, Chen and colleagues identified long-chain hydroxylated sulfatides to be selectively enriched in neoplastic epithelium of intraductal papillary mucinous neoplasms (IPMN), established precursor lesions to pancreatic ductal adenocarcinoma (PDAC) (Chen et al., 2025). Through spatial transcriptomics, the authors further showed cognate transcripts involved in sulfatide metabolism, including ceramide galactosyltransferase (also known as UGT8) and galactose-3-O-sulfotransferase 1 (GAL3ST1), to co-localize with areas of sulfatide enrichment. Mechanistically, sulfatide biosynthesis was linked to maintenance of mitochondrial morphology and function. Genetic or pharmacological suppression of UGT8 resulted in mitochondrial enlargement, loss of mitochondrial respiration, and increased susceptibility to intrinsic apoptosis (Chen et al., 2025). These findings insinuate a biophysical role of sulfatide in maintaining cancer-cell associated mitochondrial function, which may have broad relevance to other cancer types (Yoda et al., 1979; Morichika et al., 1996; Makhlouf et al., 2004).

Vykoukal and colleagues reported that enhanced scavenging of sphingomyelin by cancer cells, which they detailed in the context of triple negative breast cancer, is a ready source of ceramides for glycosphingolipid biosynthesis that supports extracellular vesicle (EV)-mediated clearance of damaged mitochondria as an alternative complementary mechanism to mitophagy. Glucosylceramide synthase (UGCG) was identified as a key mediator of this onco-metabolic process. Small molecule targeting of UGCG via repurposing of eliglustat resulted in ceramide-mediated mitophagy and subsequent cell death *in vitro* and markedly attenuated tumor development *in vivo* (Vykoukal et al., 2025). Similar findings have also been reported in prostate cancer (Vykoukal et al., 2020). These findings provide compelling evidence linking glycosphingolipid metabolism to regulation of mitochondrial dynamics, with potential therapeutic benefit.

## Metabolite-mediated modulation of the tumor microenvironment

Metabolites within the TME may emerge from cancer cells, stromal and immune cells with considerable work to-date describing the biological impact of metabolic by-products such as lactate, kynurenine, and adenosine on the TME (Chen et al., 2024; Tang et al., 2023; Zhang et al., 2026; Watson et al., 2021; Dahal et al., 2024). Additional lipid-associated metabolites such as acetate, phosphoethanolamine (pEtn), and bioactive aldehydes within the TME have since been identified to promote cancer progression.

Specifically, Murthy and colleagues demonstrated that cancer-associated fibroblasts (CAFs) release acetate to fuel pancreatic cancer (PDAC) survival under metabolic stress through acetyl-CoA synthetase short-chain family member 2 (ACSS2)-mediated acetylation of SP1 and spermidine/spermine N^1^-acetyltransferase 1 (SAT1)-drives upregulation of polyamine metabolism (Murthy et al., 2024). Genetic or pharmacological inhibition of the ACSS2-SP1-SAT1 axis attenuated tumor development in cell line- and patient-derived xenograft models of PDAC (Murthy et al., 2024). CAF-derived lactate has also been shown to induce lactylation of the spliceosome component SNRPA at Lys123 (K123), resulting in enhanced chromatin binding and androgen receptor splicing in prostate cancer cells that drives resistance to androgen-deprivation therapy (ADT) (Zhao et al., 2026). Targeting of the lactate transporter via monocarboxylate transport inhibitors effectively restored sensitivity to ADT (Zhao et al., 2026).

Wang et al. identified phosphoethanolamine (pEtn), an intermediate in phospholipid biosynthesis, to be highly elevated in tumor interstitial fluid and increased accumulation of this onco-metabolite was found to suppress CD8^+^ T-cell effector function and increase expression of inhibitory molecules PD-1, Tim-3, and Lag3 (Wang et al., 2025). Mechanistically, pEtn promoted CD8^+^ T-cell dysfunction by limiting diacylglycerol-dependent TCR signaling (Wang et al., 2025). Using a B16-*Pcyt2* overexpression model of melanoma, authors further found that reduction of intratumoral pEtn levels improved T-cell function and tumor control compared to B16-empty vector control-tumor bearing mice, supporting the notion that pEtn is an immunosuppressive onco-metabolite (Wang et al., 2025).

Beyond pEtn, lipid byproducts such as the reactive aldehyde acrolein also contribute to an immunosuppressive TME. Haku and colleagues showed that loss of HADHA and SLC25A20 impairs fatty acid oxidation, causing mitochondrial stress, acrolein accumulation, AKT-mTOR activation, GLUT1 upregulation, and T cell exhaustion. Blocking acrolein accumulation with lipid peroxidation inhibitors or N-benzylhydroxylamine enhanced PD-1 blockade efficacy in MC38 colon adenocarcinoma models (Haku et al., 2026).

## Intersection between microbial-derived metabolites and modulation of the tumor microenvironment

Seminal work by others has demonstrated that alterations in microbial metabolism directly influence tumor behavior and response to therapy, and it is now acknowledged that the symbiotic relationship between mammalian organisms and ‘microbiomes’ is a new hallmark of cancer (Ferrari and Rescigno, 2023). Bachem et al. showed that microbiota-derived butyrate enhances anti-melanoma immunity by preserving tumor-specific CD8^+^ T cell stemness in tumor-draining lymph nodes. Mechanistically, butyrate acts directly on CD8^+^ T cells and promotes a FOXO1-dependent transcriptional program, thereby increasing CD127^+^ stem-like CD8^+^ T cells while limiting terminal exhaustion. These findings suggest that microbial metabolites can enhance anti-tumor T cell immunity and improve immune checkpoint blockade efficacy (Bachem et al., 2025). Jia et al. demonstrated that *Lactobacillus johnsonii* enhances immune checkpoint blockade efficacy across cancer types by promoting stem-like CD8^+^ T cell immunity. At the molecular level, *L. johnsonii* cooperates with *Clostridium sporogenes* to generate indole-3-propionic acid, which increases H3K27 acetylation at the *Tcf7* super-enhancer and activates progenitor exhausted CD8^+^ T cells. These findings identify a microbiota-derived epigenetic metabolite axis that preserves T cell stemness (Jia et al., 2024).

This Research Topic highlights metabolic reprogramming as a key driver of tumor microenvironment remodeling and cancer progression. Metabolites derived from tumor, stromal, immune, and microbial cells reshape nutrient availability and immune function while creating exploitable metabolic vulnerabilities. Targeting these metabolic dependencies may therefore provide new therapeutic opportunities, especially when integrated with immune checkpoint inhibitors or other existing anticancer therapies to enhance treatment efficacy.
