# Correction: Editorial: Cancer cell metabolism and tumor microenvironment remodel

**DOI:** 10.3389/fgene.2026.1995063

**Published:** 2026-09-22

**Authors:** Daniela Rodriguez-Perera, Katsuhiro Yoshimura, Johannes Fahrmann, Rongzhang Dou

**Affiliations:** 1 Department of Tumor Pathology, Hamamatsu University School of Medicine, Hamamatsu, Japan; 2 Department of Clinical Cancer Prevention, The University of Texas MD Anderson Cancer Center, Houston, TX, United States

**Keywords:** cancer immune, cell metabolism, microbiome, therapy, tumor micro environment (TME)

The order of authors in the author list of the published paper was erroneously given as: Katsuhiro Yoshimura^1^*, Daniela B. Rodriguez-Perera^2^, Rongzhang Dou^2^* and Johannes Fahrmann2*.

The correct author list reads: Daniela Rodriguez-Perera 1, Katsuhiro Yoshimura^2^*, Johannes Fahrmann^1^*, and Rongzhang Dou^1^*.

The original version of this article has been updated.

